# Will cytokinins underpin the second ‘Green Revolution’?

**DOI:** 10.1093/jxb/eraa447

**Published:** 2020-12-30

**Authors:** Paula E Jameson, Jiancheng Song

**Affiliations:** 1 School of Life Sciences, Yantai University, Yantai, China; 2 School of Biological Sciences, University of Canterbury, Christchurch, New Zealand

**Keywords:** Crop, cytokinin, cytokinin oxidase/dehydrogenase (CKX), seed, isopentenyl transferase (IPT), yield

## Abstract

This article comments on:

**Schwarz I, Scheirlinck MT, Otto E, Bartrina I, Schmidt RC, Schmülling T**. 2020. Cytokinin regulates the activity of the inflorescence meristem and components of seed yield in oilseed rape. Journal of Experimental Botany **71**, 7146–7159.

**A substantial body of knowledge relating to plant growth and development has derived from research on model plants—particularly *Arabidopsis thaliana* (Arabidopsis). It is imperative that this knowledge is used to develop crop plants that are not only higher yielding but also tolerant of environmental stressors, under the joint scenario of limitations of productive land and global climate change.** Schwarz *et al.* (2020)  **provide a prime example of translational research, where knowledge from earlier Arabidopsis research with the cytokinins conducted in Thomas Schmülling’s lab (**  Bartrina *et al.*, 2011  **) has now been directly applied to increasing yield traits in *Brassica napus* L, oil seed rape.**

The gibberellins are the plant hormone that underpin the semi-dwarf cereals of the Green Revolution, with the crop plants showing either reduced response to gibberellin (wheat and maize) or reduced ability to synthesize gibberellin (rice) (Hedden, 2003). However, at the time, selections within these crops were based on high-yielding varieties with a semi-dwarf phenotype which, alongside improved resistance to lodging, had increased seed yield due to a greater proportion of assimilate being reallocated to the grain (Fischer and Stockman, 1986). In other words, seed yield *per se* was not directly selected for. Not only have the cytokinins been shown to directly increase seed yield (e.g. Ashikari *et al.*, 2005; Bartrina *et al.*, 2011; Schwarz *et al.*, 2020), they are also a significant component of the response of plants to environmental stressors (Cortleven *et al.*, 2019). Because of their recognized effects on increasing seed number and seed size and our ability to moderate internal levels of cytokinins to ameliorate the effects of stress and mineral nutrient deficiencies (reviewed in Chen *et al.*, 2020), the cytokinins may well be the hormone that underpins the second ‘Green Revolution’ (Lynch, 2007). For a brief summary of the cytokinins, please refer to Box 1.

Box 1. **The isoprenoid cytokinins**

The naturally occurring cytokinins, which are adenine derivatives, fall into two classes—those with an isoprenoid side chain and those with an aromatic side chain. The isoprenoid cytokinins are ubiquitous in the plant kingdom and are considered to be the predominant cytokinin type. Cytokinin levels within the plant are controlled by biosynthesis, translocation, destruction, and inactivation (Sakakibara, 2006). Additionally, responses to the active forms require functional receptors and signal transduction components. The cytokinins are biosynthesized either directly by ADP/ATP-isopentenyl transferases (IPTs) or indirectly by tRNA-IPTs with subsequent turnover of the isoprenylated tRNA molecules (Miyawaki *et al.*, 2006). The cytokinins exist in free base, riboside, and nucleotide forms. The nucleotides are the first formed cytokinins which can be converted by Lonely Guy (LOG) to the free base forms. The four free base forms—*trans*-zeatin (*t*Z), *cis*-zeatin (*c*Z), dihydrozeatin (DHZ), and isopentenyl adenine (iP)—are considered the active forms detected by two-component receptors. iP carries an unmodified isopentenyl side chain, DHZ has a saturated side chain, whereas *t*Z and *c*Z carry hydroxylated side chains. Cytokinin oxidase/dehydrogenase destroys cytokinin activity by removing the side chain of the *t*Z-, *c*Z-, and iP-type cytokinins. Inactivation occurs through *O*-glucosylation of the side chains of *t*Z, *c*Z, and DHZ, and their ribosides and nucleotides. As these forms can be reactivated by β-glucosidases, they are considered to be storage forms. Further inactivation can occur by glucosylation at positions 7 and 9 of the adenine moiety. Whether cytokinin levels are reduced principally through destruction by CKX or inactivation by *O*-glucosylation or *N*-glucosylation (or all three) can be species and organ specific (reviewed in Jameson, 2017).

It is the manipulation of the isoprenoid cytokinins that is the focus of much of the recent interest in the cytokinins. However, manipulation of cytokinins can activate strong internal homeostatic mechanisms, making interpretation of cytokinin levels in such plants at any developmental point in time challenging (Gasparis *et al.*, 2019; Schwarz *et al.*, 2020). Most higher plants will have more than two dozen cytokinin forms, many of which are capable of interconversion. There are relatively few laboratories equipped with both the knowledge and instrumentation required for comprehensive cytokinin analyses. There are no shortcuts: measuring and reporting on cytokinins detected in crude or even partially purified extracts by immunoassay, as exemplified in a recently published paper, in which the ‘cytokinin’ detected was not even named (Tsago *et al.*, 2020), is simply unacceptable.

While the cytokinins had, for many years, been implicated in regulating seed yield (reviewed in Jameson and Song, 2016), it was the seminal work of Ashikari *et al.* (2005) that cemented their importance in crop production. They showed that rice cultivars with mutations in *CKX2*, which codes for the enzyme that destroys cytokinin, had increased seed number. The work with rice was followed by that of Bartrina *et al.* (2011) showing that double *ckx3,5* mutants of Arabidopsis had increased seed number. Subsequently, numerous approaches have supported a role for *CKX* in negatively controlling seed number and seed size (reviewed in Chen *et al.*, 2020).

## Amelioration of drought stress

One of the most significant challenges when working with the cytokinins is that they have opposite effects on shoot and root growth—promoting shoot growth while inhibiting root growth (Werner *et al.*, 2003)—so that a generic increase in cytokinin levels may well promote shoot growth while inhibiting root growth. Cytokinins also delay senescence, but too much cytokinin may lead to a competitive interaction between source leaves and the sinks (pods and seeds) (e.g. Sýkorová *et al.*, 2008), or even cause excessive branching through the release of apical dominance (McKenzie *et al.*, 1998). Increased cytokinin also leads to an increase in tillering in cereals, which may in fact not be ideal if the tillers mature at different stages (Chen *et al.*, 2020). Significantly, however, modest enhancement of cytokinin levels, through careful selection of senescence-, maturation-, or stress-responsive promoters to drive the biosynthetic isopentenyl transferase (*IPT*) gene, has been a successful strategy to ameliorate the impacts of drought stress on yield of both dicots and monocots, including under field conditions (reviewed in Jameson and Song, 2016; Joshi *et al.*, 2019).

The alternative approach to manipulating cytokinin levels *in planta* has been either to increase the expression of *CKX* in the roots or to decrease the expression of *CKX* in the shoots. By targeting the expression of specific *CKX* gene family members to the roots, using root-specific promoters, the inhibitory cytokinin levels can be reduced, root growth promoted, drought tolerance enhanced, and nutrient accumulation increased (Werner *et al.*, 2010; Ramireddy *et al.*, 2018; reviewed in Chen *et al.*, 2020). Interestingly, Schmülling’s lab recently showed that constitutive (rather than root-specific) expression of *CKX2* in oil seed rape also led to increased root growth without any impact on shoot growth, enhanced drought tolerance, and the accumulation of essential micronutrients in the leaves (Nehnevajova *et al.*, 2019). It will be interesting to know the effect on root growth and drought resilience of the *ckx3,5* oil seed rape mutant developed by Schwarz *et al.* (2020).

## Targeted manipulation of seed yield

As mentioned above, the Os*CKX2* mutant of rice (Ashikari *et al.*, 2005), and the double *ckx3,5* mutants of Arabidopsis (Bartrina *et al.*, 2011) and oil seed rape (Schwarz *et al.*, 2020), had increased yield traits (see also Chen *et al.*, 2020). In contrast, targeted reduction through using RNAi and, more recently, gene editing, has not always yielded the results that experiments with mutants might have led us to expect. For instance, Gasparis *et al.* (2019) used gene editing to knock out barley *CKX1* or *CKX3*. The results obtained were not straightforward and indicated that strong cytokinin homeostatic mechanisms came into effect, leading to different morphological and somewhat unanticipated responses for each of the two gene-edited lines. They concluded that the effect of full knockout, compared with RNAi knockdown (e.g. Zalewski *et al.* 2014), may have led to a strong homeostatic response. They suggested that knockdown of two *CKX* gene family members may be required, as was required in both Arabidopsis and oil seed rape, although not in rice (Ashikari *et al.*, 2005).

## Combining the old and new: mutagenesis and next-generation sequencing

The controlled expression of *IPT* and the targeting of *CKX* via RNAi or gene editing all require transformation and cannot be readily scaled up. Furthermore, transgenic plants require regulatory clearance in many countries, and cannot be commercially grown in some jurisdictions (Fritsche *et al.*, 2018). On the other hand, mutagenesis has been an aid to plant breeding for >80 years and plants derived through mutagenesis are free from regulatory hurdles. Traditionally, mutagenesis breeding has been carried out on a large scale through painstaking phenotypic selection and backcrossing over multiple generations. Now, the combination of TILLING (Targeting Induced Local Lesions in Genomes), which provides a non-transgenic method of inducing point mutations into a genome using ethyl methanesulfonate (EMS) as the mutagen, with whole-genome or exome sequencing, enables the identification of mutations in multiple lines (Uauy *et al.*, 2017; Chen *et al.*, 2020). Once a specific mutation is detected, it is important to recognize that four to five generations of backcrossing to the parent is still required to provide a resource enriched in that mutation. However, with the use of the ‘speed breeding’ strategy, several generations per year can be accommodated (Ghosh *et al.*, 2018), which is similar to the number of generations that can be achieved with model plants such as Arabidopsis—making working with non-model species that much more accessible.

Based on the previous work in Thomas Schmülling’s lab on Arabidopsis (Bartrina *et al.*, 2011), Schwarz *et al.* (2020) focused on the *CKX* gene family members *3* and *5*. However, oil seed rape is tetraploid, making it necessary to identify the EMS-induced mutations in each of the four copies of *ckx3* and the two copies of *ckx5* in the *B. napus* genome. Four generations of backcrosses were done for each of the six individually identified mutations, before the lines were crossed to achieve the quadruple *ckx3* mutant, the double *ckx5* mutant, and, finally, the sextuple *ckx3,5* mutant which has a combination of all six of the individual loss-of-function alleles. Only following this could Schwarz *et al.* (2020) characterize the sextuple mutant which they did comprehensively both phenotypically and microscopically, along with its transcriptome and changes in cytokinins. While some differences between the Arabidopsis *ckx3,5* and the sextuple *ckx3,5* oil seed rape mutants were apparent, the translation of the information from the model plant to the crop plant strongly indicated that *CKX* has an evolutionarily conserved role in controlling seed yield (Bartrina *et al.*, 2011; Schwarz *et al.*, 2020). Further, they suggest that simultaneous enhancement of source strength (possibly via controlled *IPT* expression) with the increased sink capacity of the *ckx3,5* mutant may provide resources to fill the additional ovules that were formed but did not mature.

The challenges that faced Thomas Schmülling’s group with their tretraploid *B. napus* are exacerbated in crops including bread wheat, which is hexaploid. Here, the cytokinins, and the *CKX* gene family members in particular, are key targets (Chen *et al.*, 2020; Jabłoński *et al.*, 2020). Utilizing TILLING, speed breeding, and whole-exome sequencing, Song *et al*. (unpublished data) have identified multiple point mutations for all of the *CKX* gene family members from the A, B, and D subgenomes in the bread wheat genome (IWGSC Ref Seq 2.0). The phenotype and contribution to seed yield of each family member will be evaluated after the triple ABD mutants are stacked and serial backcrossing to the wild type has been undertaken.

With our ability to enhance both source (leaf) and sink (pods and seeds) activities, as well as to ameliorate the impacts of stress (roots, leaves), and enhance nutrient uptake, through moderating cytokinin biosynthesis (IPT) and destruction (CKX), the cytokinins are indeed poised to drive the second ‘Green Revolution’ (Box 2).

Box 2. **Effect on yield traits of modifying cytokinin content in leaf, shoot apical meristem, seed, and root**

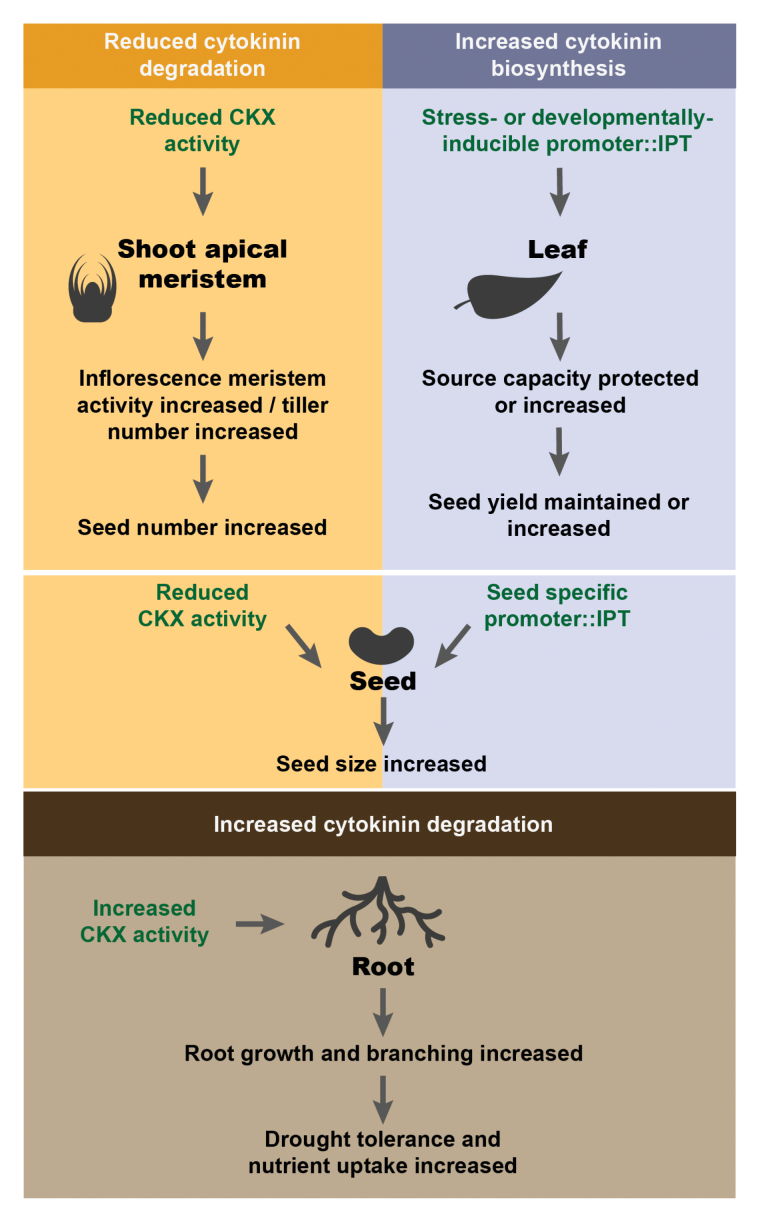

IPT, isopentenyl transferase; CKX, cytokinin oxidase/dehydrogenase.

Figure adapted from Jameson and Song. 2016.
